# The Leaf Economics Spectrum Constrains Phenotypic Plasticity Across a Light Gradient

**DOI:** 10.3389/fpls.2020.00735

**Published:** 2020-06-11

**Authors:** Xiaoping Chen, Jun Sun, Mantang Wang, Min Lyu, Karl J. Niklas, Sean T. Michaletz, Quanlin Zhong, Dongliang Cheng

**Affiliations:** ^1^Fujian Provincial Key Laboratory of Plant Ecophysiology, Fujian Normal University, Fuzhou, China; ^2^Key Laboratory of Humid Subtropical Eco-geographical Process, Ministry of Education, Fuzhou, China; ^3^School of City and Architecture Engineering, Zaozhuang University, Zaozhuang, China; ^4^Plant Biology Section, School of Integrative Plant Science, Cornell University, Ithaca, NY, United States; ^5^Department of Botany and Biodiversity Research Centre, University of British Columbia, Vancouver, BC, Canada

**Keywords:** leaf functional traits, within-canopy, plasticity, sun and shade-leaves, convergent LES relationships

## Abstract

The leaf economics spectrum (LES) characterizes multivariate correlations that confine the global diversity of leaf functional traits onto a single axis of variation. Although LES is well established for traits of sun leaves, it is unclear how well LES characterizes the diversity of traits for shade leaves. Here, we evaluate LES using the sun and shade leaves of 75 woody species sampled at the extremes of a within-canopy light gradient in a subtropical forest. Shading significantly decreased the mean values of LMA and the rates of photosynthesis and dark respiration, but had no discernable effect on nitrogen and phosphorus content. Sun and shade leaves manifested the same relationships among *N*_mass_, *P*_mass_, *A*_mass_, and *R*_mass_ (i.e., the slopes of log–log scaling relations of LES traits did not differ between sun and shade leaves). However, the difference between the normalization constants of shade and sun leaves was correlated with functional trait plasticity. Although the generality of this finding should be evaluated further using larger datasets comprising more phylogenetically diverse taxa and biomes, these findings support a unified LES across shade as well as sun leaves.

## Introduction

A central premise of trait-based ecology is that performance and functioning across levels of organization, from organs to ecosystems, are predictable from organ-level functional traits (Niklas, 1994; Westoby et al., 2002; Hallik et al., 2009; Adler et al., 2014; Wullschleger et al., 2014; Fisher et al., 2015; Christoffersen et al., 2016; Díaz et al., 2016; McDowell et al., 2018). The leaf economics spectrum (LES) offers substantial potential for quantifying the broad diversity of leaf functional traits to predict changes in community composition in response to global change, as well as how these changes in composition will feedback to ecosystem function (Reich et al., 1998; Wright et al., 2004). However, the data used to construct the LES is primarily based on the functional traits of sun leaves (i.e., leaves exposed to direct sunlight) and it is unclear whether the trends established by the LES extend to shade leaves.

The LES characterizes evolutionary convergence on robust and consistent relationships among the form, function, chemistry, and longevity of leaves from diverse ecosystems and climates, confining this diversity onto a single axis of variation. Specifically, the LES describes trade-offs among leaf mass per area (LMA), nitrogen and phosphorus contents per unit mass (*N*_mass_ and *P*_mass_, respectively), assimilation and respiration rates per unit mass (*A*_mass_ and *R*_mass_, respectively), and a number of other critically important functional traits that characterize plant physiology and ecology. The LES is shaped by the joint effects of environmental filtering and biophysical constraints on leaf carbon economics, yielding emergent functional tradeoffs between assimilation rate, leaf longevity, and leaf construction costs (Chabot and Hicks, 1982; Westoby et al., 2002; Kikuzawa and Lechowicz, 2006; Michaletz et al., 2015, 2016; Anderegg et al., 2018). These trade-offs have been demonstrated primarily using global-scale data, and thereby provide a general mechanistic framework for understanding and predicting variations of plant community structure and function across resource availability gradients. For example, under low-light conditions, the LES predicts that leaves with higher LMA will have lower *N*_mass_, *P*_mass_, and *A*_mass_, but longer life spans (Reich et al., 1998; Wright et al., 2004). At the other end of the spectrum, leaves exposed to high light conditions are predicted to have lower LMA and higher *N*_mass_, *P*_mass_, *R*_mass_, and *A*_mass_, but shorter lifespans (Chabot and Hicks, 1982; Mooney and Gulmon, 1982; Merino et al., 1984; Wright et al., 2004; Kikuzawa et al., 2013; Reich, 2014).

Despite the apparent broad applicability of the LES, a major concern is that its predictions are primarily based on data derived from sun leaves (e.g., Wright et al., 2004; Keenan and Niinemets, 2016). As noted previously, it remains uncertain as to whether the trends manifest in the LES hold equally well for shade leaves. Light availability varies more than 50-fold within dense canopies and more than 10-fold within open canopies (Hirose et al., 1997; Koike et al., 2001; Rambal, 2001; Valladares, 2003; Niinemets and Anten, 2009; Niinemets et al., 2015). Light plays a key role in leaf growth and physiology. One of the most profound effects of light on plant development is the suppression of the expression of a series of light-responsive genes (Li et al., 1995). Therefore, sun leaves might exhibit better trait full genotypic expression than shade leaves. This might be one of the reasons for why large-scale LES analyses avoided shade leaves. It is crucial to evaluate whether LES expands to shade leaves, including those from low light environments commonly found in within-canopy light gradients.

Furthermore, as formalized by the carbon economics theory (Chabot and Hicks, 1982; Blonder et al., 2011; Michaletz et al., 2015, 2016), leaf functional traits are expected to vary in response to within-canopy light gradients in order to maximize leaf carbon return on investment. Indeed, it has long been known that many leaf functional traits exhibit plasticity and/or acclimation across within-canopy light gradients (Iio et al., 2005; Lloyd et al., 2010; Kattge et al., 2011; Kenzo et al., 2015). For example, LMA and nitrogen content per area generally decrease with decreasing irradiance (Hirose and Werger, 1987; Givnish, 1988; Hollinger, 1996; Pons and Anten, 2004; Posada et al., 2009; Fajardo and Siefert, 2016), which helps to maximize leaf net carbon gain because of an accompanied lower carbon cost (Anten, 2016). In general, because of the reallocation of nitrogen, leaf N content (the “investment”) along light gradients within plants does not agree with theoretical expectations that N declines with decreasing light availability from the top toward the bottom of the canopy (Hirose and Werger, 1987; Givnish, 1988; Anten, 2016). Hence, large light-dependent sensitivity in LMA appears to be responsible for the observed differences in the photosynthetic production and nutrient content of woody plants (Niinemets et al., 2015; Messier et al., 2016). However, relatively little attention has been paid to LES of shade leaves. Using a worldwide database (spanning 167 species with LMA measurements, 75 species with *N*_mass_ measurements, and 44 species with *A*_mass_ measurements), Keenan and Niinemets (2016) concluded that LES, thought to comprise data for sun leaves only, might instead include a large proportion of data for leaves growing under shaded conditions, implicitly suggesting that LES may apply generally to all leaves.

Each trait underlying the LES may be affected by canopy conditions and exhibit plasticity (Messier et al., 2010; Sandel et al., 2010). This suggests that values of a certain LES trait in sun and shade leaves might be consistently correlated across all species but that the scaling exponents and normalization constants of the relationship might differ numerically (Wright et al., 2001; Wright and Sutton-Grier, 2012; Niklas and Hammond, 2019). For example, the numerical values of the scaling exponents of some LES relationships have been shown to differ for plants growing in environments with contrasting water and nutrient availabilities (Wright et al., 2001). In addition, Wright and Sutton-Grier (2012) have suggested that trends in the traits of individual species do not consistently predict those of other species. Therefore, it is unclear whether the scaling exponents and normalization constants of LES trait-trait relationships are consistent between sun and shade leaves.

Here, we evaluate the effects of within-canopy shading on LES. Specifically, we examine relationships among five key leaf functional traits (i.e., LMA, *N*_mass_, *P*_mass_, *A*_mass_, and *R*_mass_) to address two key questions. First, to what degree are leaf functional traits plastic between sun- and shade- leaves? Second, do LES relationships exist for both sun- and shade-leaves? And, if they do, are the scaling relationships between sun and shade leaves consistent? We hypothesize that (i) shade induced morphological plasticity is a stronger determinant of leaf acclimation to light availability compared to chemical (*N*_mass_ and *P*_mass_) or physiological plasticity (*A*_mass_ and *R*_mass_) (see Vaz et al., 2010; Messier et al., 2016); and (ii) LES relationships should hold for shade leaves, but the numerical values of the scaling exponents and normalization constants of some trait scaling relationships may differ between sun and shade leaves.

## Materials and Methods

### Study Site

This study was conducted in the Yangjifeng National Nature Reserve of Jiangxi, southeastern China (117°11′30″∼117°28′40″ E, 27°51′10″∼ 28°02′20″ N, 1540.9 m a.s.l.). The reserve has a typical humid mid-subtropical monsoon climate. The range of annual precipitation average is 1870–2191 mm, 72–75% of which falls from April to June. The range of average annual temperature is 11.4–18.5°C. The soil is a haplic lixisol. In August 2015, a 500 m × 500 m plot was established. The tree density was 703.5 trees ha^–1^, mean diameter at breast height (DBH) was 11.77 ± 0.10 cm (mean ± SE), mean height was 8.54 ± 0.06 m, mean crown base height was 2.85 ± 0.02 m, and mean crown diameter was 3.18 ± 0.02 m. The dominant species were *Castanopsis fargesii*, *Alniphyllum fortune*, *Litsea cubeba*, *Castanopsis carlesii*, *Elaeocarpus sylvestris*, and *Schima superba*.

### Gas-Exchange Measurements

Branches for gas exchange measurements were collected in August 2017. Seventy-five species, from 61 genera and 33 families, were selected based on abundance (representing 88.12% of individuals with DBH ≥ 1 cm in the plot; Supplementary Table S1). Three individuals were sampled from each species. To minimize the confounding effects of leaf age (Wright et al., 2006) and branch length (Niklas and Cobb, 2010), three current-year branches (twigs) were selected from each of the canopy top (sun leaves) and canopy bottom (shade leaves) for each individual following the protocol of Sack et al. (2006). Branches were collected from canopies and immediately placed in water to reduce water loss. Embolisms were removed by re-cutting branch ends under water (Yoder et al., 1994; Mori et al., 2010; Michaletz et al., 2016; Kelly et al., 2017; Malhi et al., 2017). There were no significant differences between branch traits from sun and shade canopy positions (*P* > 0.05, *t*-tests) (Supplementary Table S2). For each sampled tree, the photosynthetic photon flux densities (PPFDs) of leaves at the sampled canopy locations were measured on a sunny day with a LI-250A light meter (LICOR, Lincoln, NE, United States). All the PPFDs of the sun leaves (1172.25 ± 25.73 μmol m^–2^ s^–1^) were significantly higher than that in the shade leaves (82.88 ± 3.23 μmol m^–2^ s^–1^) (Supplementary Figure S1A). The PPFDs at the shade leaves consisted of about 7.15% ± 0.36 (Supplementary Figure S1A) of full sunlight in the sun leaves.

Gas-exchange measurements were conducted on six to nine leaves from each branch. Leaves showing no evidence of disease or herbivory were used for all measurements and kept hydrated throughout the time measurements were taken and recorded. Maximum net assimilation rates (*A*_area_; μmol m^–2^ s^–1^) and respiration rates (*R*_area_) were estimated from light response curves obtained using a LI-6800 portable photosynthesis system and calculated by Photosynthesis (LICOR, Lincoln, NE, United States). During measurements, leaf temperatures were maintained at 25°C, chamber CO_2_ was maintained at 400 p.p.m, and flow rates were maintained at 500 mmol s^–1^. To obtain light response curves, net assimilation rates were measured at PPFDs of 2000, 1600, 1200, 900, 600, 300, 200, 100, 80, 50, 20, and 0 μmol m^–2^ s^–1^ (Supplementary Figure S1B).

### Leaf Mass per Area, Leaf Nitrogen, and Leaf Phosphorus Content Measurements

All of the leaves from each branch were detached and scanned using a flatbed scanner (Epson V39, Epson, Japan), yielding a total of data from 7417 leaves. Leaf area was measured from scanned images using Image J (National Institute of Health, Bethesda, ML, United States). Leaf samples were dried subsequently at 75°C for 48 h before measuring dry weight. Samples were then ground and passed through a 100-mesh sieve (0.15 mm). The leaf nitrogen content per unit mass (*N*_mass_,%) was determined using an Element Analyzer (VARIO EL III Element Analyzer, Elementar, Germany). The leaf phosphorus content per unit mass (*P*_mass_,%) was measured using the molydate/ascorbic acid method and a continuous flow analyzer (SKALAR SAN++, Netherlands) after H_2_SO_4_-HClO_4_ (4:1, v:v) digestion.

### Data Analysis

#### Leaf Trait Plasticity

To evaluate whether the interspecies traits of sun leaves are significantly different than those of shade leaves, traits were compared using *t*-tests and linear regression analysis. For each trait, an index of maximum within-canopy plasticity was calculated as the quotient for sun leaves divided by that of shade leaves following the protocol of Sack et al. (2006) after calculating the average of a trait in sun and shade leaves at three taxonomic levels (i.e., species, genus, and family). Linear mixed effect models were fit using the lme4 packages in R (v.3.5.1; R Foundation for Statistical Computing, Vienna, Austria) to determine the extent to which the three different taxonomic levels contributed to trait variation. Analyses showed that there were no significant differences of leaf trait plasticity among the three taxonomic levels (Table 1 and Supplementary Table S3) and that among-individual and among-species differences together consistently explained over 50% of the total trait variance with the exception LMA (i.e., 48.57%) (Supplementary Figure S2). Thus, leaf trait plasticity was expressed as the average plasticity of all species.

**TABLE 1:** Summary of traits for sun and shade leaves from 75 tree species in a subtropical forest.

Traits	Sun-leaves		Shade leaves		Plasticity	
			
	Range	Mean ± SE	Range	Mean ± SE	Range	Mean ± SE
LMA (g m^–2^)	38.22–153.23	83.68 ± 3.20*	31.31–141.29	75.25 ± 2.60*	0.62–2.35	**1.13 ± 0.03^c^**
*A*_mass_ (nmol g^–1^ s^–1^)	20.58–291.22	113.79 ± 6.51*	21.89–177.83	66.51 ± 3.71*	0.90–3.82	**1.80 ± 0.07^a^**
*R*_mass_ (nmol g^–1^ s^–1^)	1.44–37.08	9.50 ± 0.78*	1.21–26.04	6.25 ± 0.50*	0.64–5.53	**1.63 ± 0.09^b^**
*N*_mass_ (%)	0.83–3.68	2.04 ± 0.07	0.89–3.53	1.99 ± 0.06	0.89–3.53	**1.02 ± 0.01^d^**
*P*_mass_ (%)	0.05–0.20	0.11 ± 0.004	0.05–0.20	0.12 ± 0.004	0.78–1.50	**0.98 ± 0.02^d^**

*Significant differences are denoted in bold; asterisks indicate significant differences in trait means between sun and shade leaves, while superscripted letters indicate significant differences in mean plasticity among traits (t-test, *P* < 0.05).*

#### PCA Analysis

To check how sun and shade leaf traits variability is organized, a principle component analysis (PCA) was conducted using species as data points. All variables were log-transformed before analysis to improve normality. PCAs were performed for the five traits using the ape (Paradis et al., 2004) and vegan (Dixon, 2003) packages in R (v.3.5.1; R Foundation for Statistical Computing, Vienna, Austria). Bivariate correlations between each trait and PCA axis 1 (PC1) and axis 2 (PC2) were evaluated using Pearson’s correlation coefficient (*r*).

#### Pairwise Scaling Relationships

We examined bivariate scaling relationships between LMA, *N*_mass_, *P*_mass_, *A*_mass_, and *R*_mass_, and compared our results with those of a published dataset of global trait variation (Wright et al., 2004). The formula *y* = β*x*^α^ (Equation 1) was used to examine bivariate trait relationships. To linearize this relationship, Equation (1) was log-transformed to give the linear form log (*y*) = log (β) + α log (*x*) (Equation 2), where *y* and *x* are interdependent leaf functional traits of interest, β is the normalization constant, and α is the scaling exponent (the slope of the log-log linear bivariate plot). The linear formula was then fit to data using model II standardized major axis (SMA) regression (Warton et al., 2012) using the smatr package 3.4-3 in R (v.3.5.1; R Foundation for Statistical Computing, Vienna, Austria). When no significant numerical differences between two or more scaling exponents (*P* > 0.05) for comparable bivariate comparisons were observed (e.g., sun vs. shade leaves), a common scaling exponent was then estimated.

## Results

### Variation of Traits for Sun and Shade Leaves

**FIGURE 1:**
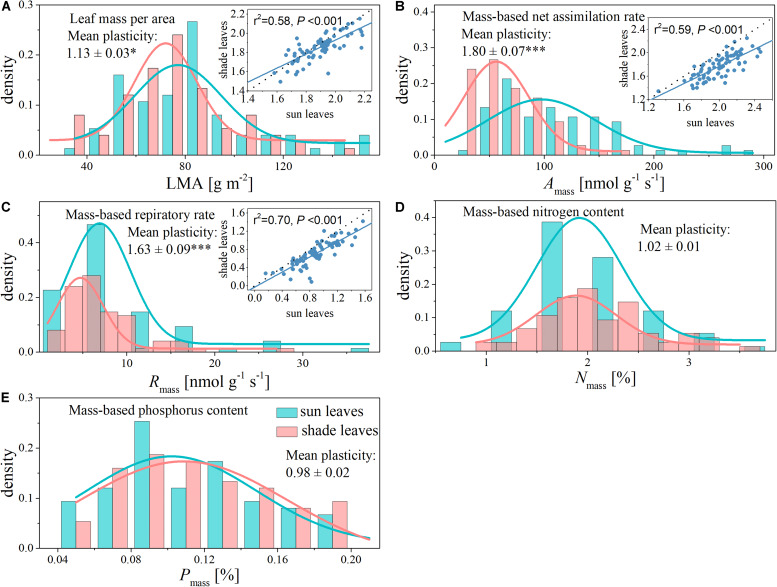
Histogram with normal distribution and shade-sun trait relationships for functional traits of sun and shade leaves from the same canopy. The dashed lines indicate 1:1. **(A)** Leaf mass per area (LMA); **(B)** mass-based net assimilation rate (*A*_mass_); **(C)** mass-based respiratory rate (*R*_mass_); **(D)** mass-based nitrogen content (*N*_mass_); **(E)** mass-based phosphorus content (*P*_mass_). *indicates a significant difference at *P* < 0.05 and ***indicates a significant difference at *P* < 0.001.

Within a given canopy location (sun or shade), we observed more than one order of magnitude variation in each of the five leaf traits (Table 1). The most variable trait was *A*_mass_, with a 14-fold range in sun leaves and a 7-fold range in shade leaves (Table 1 and Figure 1). In contrast, the least variable trait was *N*_mass_, with a 4-fold range in sun leaves and a 1-fold range in shade leaves (Table 1 and Figure 1).

Shading had different effects on each of the leaf functional traits (Table 1 and Figure 1). The LMA, *A*_mass_, and *R*_mass_ of shade leaves were significantly lower compared with those of sun leaves (Figure 1). Specifically, the mean values of *A*_mass_ and *R*_mass_ respectively were 113.79 nmol g^–1^ s^–1^ and 9.50 nmol g^–1^ s^–1^ in sun leaves, 66.51 nmol g^–1^ s^–1^ and 6.25 in nmol g^–1^ s^–1^ in shade leaves (Table 1). The plasticity of *A*_mass_ and *R*_mass_ was 1.80 ± 0.07 and 1.63 ± 0.09, respectively. The mean value of LMA decreased from 83.68 g m^–2^ in sun leaves to 75.25 g m^–2^ in shade. The plasticity for LMA was 1.13 ± 0.03 (Table 1 and Figure 1). In contrast, no significant differences between sun and shade leaves were detected for *N*_mass_ and *P*_mass_, although the plasticity of *N*_mass_ (i.e., 1.02 ± 0.01) was somewhat greater than that of *P*_mass_ (i.e., 0.98 ± 0.02) (Table 1).

### PCAs of Sun and Shade Leaf Traits

**FIGURE 2:**
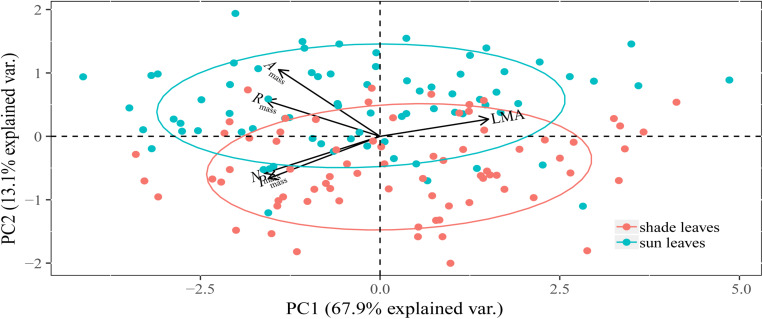
Principal component analysis (PCA) of morphological, chemical, and physiological traits of sun and shade leaves for 75 woody species. Sun and shade group was separated by ellipsoid at 68% normal probability. The PC1 scores between sun and shade leaves were not significantly different (*P* > 0.05).

The PC1 axis captured 73.32 and 69.24% of the total variation in the five traits of sun and shade leaves, respectively, with all traits contributing substantially to this axis (Table 2). The PC1 and PC2 axes captured 81.04% of the total variability across all species (Table 2 and Figure 2). When sun and shade leaves were combined, PC1 captured 67.90% of the total variation in the five leaf traits, with all traits contributing substantially to this axis (Table 2 and Figure 2).

**TABLE 2:** Bivariate relationships between individual traits and the scores of the first and second PC for sun leaves, shade leaves and whole-canopy economic spectra.

Canopy location		Explained variation (%)	logLMA	log*N*_mass_	log*P*_mass_	log*A*_mass_	log*R*_mass_
sun leaves	PC1	73.32	0.86**	−0.86**	0.86**	−0.81**	−0.89**
	PC2	10.84	−0.25*	−0.40**	−0.39**	0.38**	0.16
shade leaves	PC1	69.24	0.85**	−0.87**	−0.83**	−0.80**	−0.82**
	PC2	11.03	−0.20	−0.34**	−0.45**	0.37**	0.25*
All leaves	PC1	67.90	0.82**	−0.88**	−0.85**	−0.76**	−0.87**
	PC2	13.14	0.17	0.37**	0.40**	−0.49**	0.18

Significant correlations are denoted in bold with asterisks; * indicates a significant correlation at *r* < 0.05 and ** indicates a significant correlation at *r* < 0.001.

All traits contributed substantially to the PC1 axis (Table 2). Leaf *N*_mass_ and *P*_mass_ and physiological rates were negatively correlated with PC1, whereas LMA was positively correlated with PC1 (Table 2).

### Scaling Relationships of Sun and Shade Leaf Traits

**FIGURE 3:**
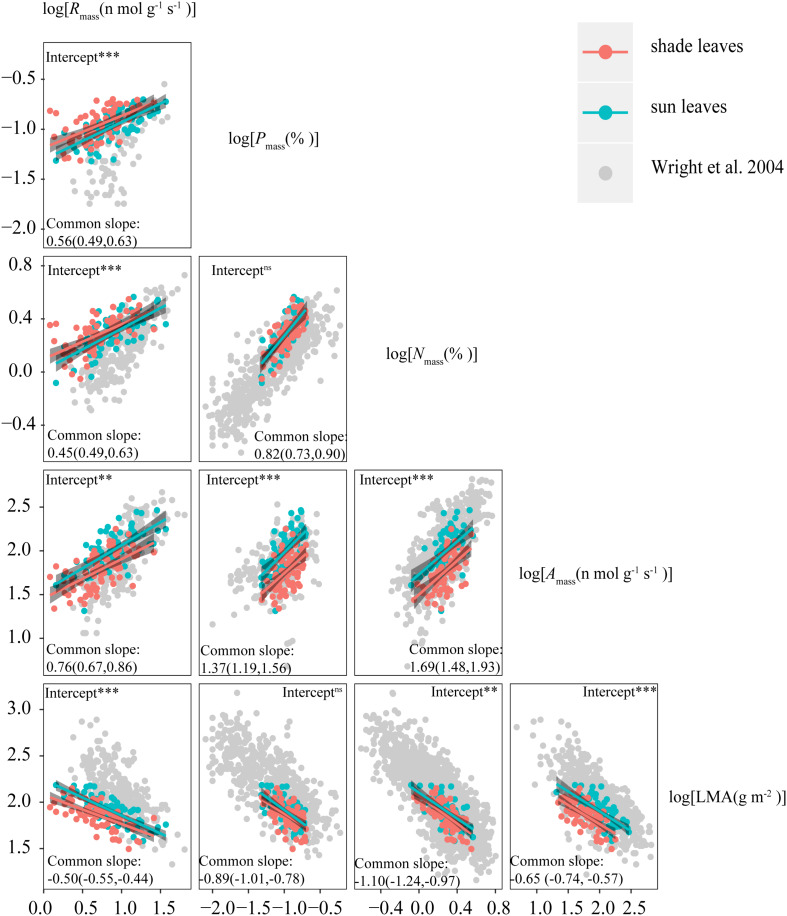
Leaf Economics Spectrum (LES) scaling relationships for sun and shade leaves from this study compared with those from the global dataset of Wright et al. (2004). The numbers in parenthesis indicate 95% confidence intervals of scaling exponents. ns indicates no significant difference between intercepts of sun and shade leaves, ** indicates a significant difference at *P* < 0.01, and *** indicates a significant difference at *P* < 0.001.

Consistent with sun leaves, robust correlations among leaf functional traits were also observed in shade leaves (Table 3, 4). With the exception of LMA vs. *N*_mass_ and LMA vs. *P*_mass_, the numerical values of scaling exponents were significantly different from ± 1.0 (Table 4), indicating that traits manifest allometric rather than isometric scaling relationships. The scaling exponents of all the bivariate trait relationships did not differ significantly between the sun and shade leaves (Table 4 and Figure 3). Indeed, generalizable scaling relationships were evident within each of the bivariate trait relationships. In the particular case of photosynthetic rates, the scaling exponents governing the N content for sun and shade leaves were 1.38 and 2.03 (common slope = 1.67), and 1.41 and 2.07 (common slope = 1.71), respectively (Table 3 and Figure 3). However, shading decreased the numerical values of the normalization constants for physiological metabolic rates vs. chemical traits and LMA scaling relationships, as well as decreased the numerical values of the normalization constants of *A*_mass_ and *R*_mass_ (Table 4 and Figure 3). In contrast, the normalization constants between *N*_mass_ (and *P*_mass_) vs. LMA as well as *N*_mass_ vs. *P*_mass_ were unaffected for leaves collected in the shade (Table 4 and Figure 3).

**TABLE 3:** Scaling exponents for bivariate relationships among mass-based traits of sun or shade leaves.

Canopy location	Leaf traits	log LMA	log *A*_mass_	log *N*_mass_	log *P*_mass_	log *R*_mass_
Sun leaves	log LMA		−0.65 (−0.77, −0.55)	−1.09 (−1.30, −0.91)	−0.91 (−1.09, −0.75)	−0.50 (−0.58, −0.43)
	log *A*_mass_	0.44 (75)		1.67 (1.38, 2.03)	1.39 (1.16, 1.68)	0.77 (0.65, 0.91)
	log *N*_mass_	0.41 (75)	0.31 (75)		0.83 (0.72, 0.96)	0.46 (0.39, 0.55)
	log *P*_mass_	0.38 (75)	0.35 (75)	0.62 (75)		0.55 (0.47, 0.66)
	log *R*_mass_	0.59 (75)	0.46 (75)	0.45 (75)	0.46 (75)	
Shade leaves	log LMA		−0.65 (−0.78, −0.54)	−1.11 (−1.32, −0.94)	−0.87 (−1.05, −0.72)	−0.49 (−0.58, −0.41)
	log *A*_mass_	0.40 (75)		1.71 (1.41, 2.07)	1.33 (1.10, 1.62)	0.75 (0.62, 0.90)
	log *N*_mass_	0.45 (75)	0.32 (75)		0.78 (0.67, 0.91)	0.44 (0.36, 0.53)
	log *P*_mass_	0.32 (75)	0.30 (75)	0.56 (75)		0.56 (0.46, 0.68)
	log *R*_mass_	0.41 (75)	0.36 (75)	0.36 (75)	0.33 (75)	

*Standardized major axis exponents with 95% confidence intervals are given in the upper right hand of the matrix (y variable is column 1, x variable in row 1). Coefficients of determination (r^2^) and sample sizes (n, in brackets) are given in the lower left hand of the matrix. All relationships are highly significant, P < 0.001.*

**TABLE 4:** Scaling exponents for bivariate relationships among mass-based traits of sun and shade leaves.

Leaf traits	log LMA	log *A*_mass_	log *N*_mass_	log *P*_mass_	log *R*_mass_
log LMA		−0.65 (−0.74, −0.57)	−1.10 (−1.24, −0.97)	−0.89 (−1.01, −0.78)	−0.50 (−0.55, −0.44)
log *A*_mass_	(150)***		1.69 (1.48, 1.93)	1.37 (1.19, 1.56)	0.76 (0.67, 0.86)
log *N*_mass_	(150)**	(150)***		0.81 (0.73, 0.90)	0.45 (0.40, 0.51)
log *P*_mass_	(150) ns	(150)***	(150)ns		0.56 (0.49, 0.63)
log *R*_mass_	(150)***	(150)**	(150)***	(150)***	

The common standardized major axis exponents with 95% confidence intervals are given in the lower left section of the matrix (y variable is column 1, x variable in row 1; see Equation 1). * indicates a significant difference at *P* < 0.05, ** indicates a significant difference at *P* < 0.01, and *** indicates a significant difference at *P* < 0.001. Sample sizes (n) are given in brackets in the lower left hand of the matrix.

**FIGURE 4:**
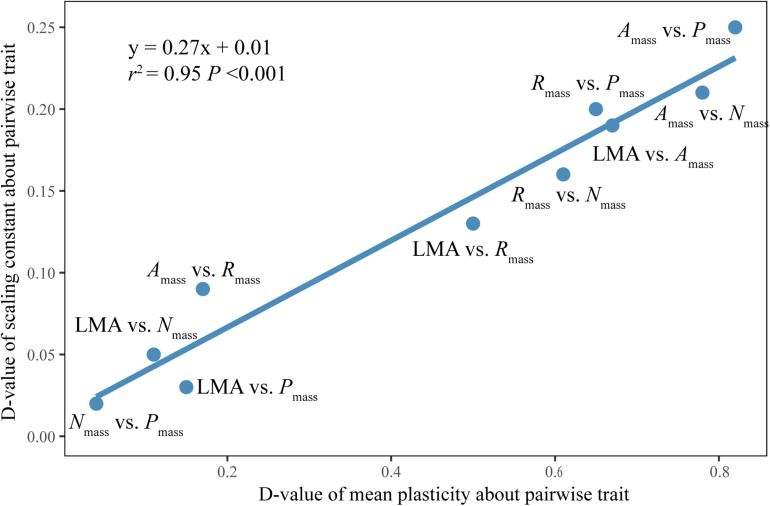
Bivariate plot of the difference of the normalization constants between sun and shade leaves against the difference of plasticity of two related functional traits. *D*-value: the difference value.

The difference of the normalization constants between sun and shade leaves was significantly positively correlated with the differences of plasticity of the related functional traits (Figure 4).

## Discussion

The goal of this paper was to evaluate the universality of LES relationships in the context of within-canopy light gradients. This goal was approached in two ways. First, we examined the leaf functional trait plasticity for sun and shade leaves. Second, we evaluated whether species-level differences in trait plasticity affected the canonical correlations predicted by the LES. In general, our results demonstrate only minimal to moderate variation in traits across leaves collected from different canopy light gradients (i.e., the relationships between sun and shade leaves scaling relationships do not statistically significantly differ), indicating that phenotypic trait variation across species is constrained by convergent leaf economic spectra.

We observed moderate levels of leaf functional trait plasticity across species within-canopy light gradients, indicating that leaf functional traits across a wide range of light conditions appear to be in equilibrium with the environment. These results are consistent with those of previous studies. For example, Sack et al. (2003) reported a limited LMA plasticity for mixed *Quercus* forests in southern Spain (i.e., average within-canopy plasticity was 1.5 or less). Wright et al. (2006) quantified the leaf N and P contents for 28 woody species along vertical canopy profiles and found that *P*_mass_ varied in a manner consistent with optimal allocation, whereas LMA and *N*_mass_ decreased with canopy depth in only two and five species, respectively. Iio et al. (2005) found that there was no significant difference in *N*_mass_ between the best-lit and the most shaded leaves within the crown of a *Fagus crenata* tree. We also found that shading had the most influence on leaf physiological traits (*A*_mass_ and *R*_mass_) followed by morphological traits (i.e., 1.13 ± 0.04 times), but shading had no disenable effect on chemical (N and P) traits (Table 1). We suggest that these modest phenotypic patterns may reflect adaptations to maximize photosynthetic carbon gain.

As predicted, regardless of whether the data were drawn from sun or shade leaves, species at the fast-return end of the LES had high leaf nutrient contents, high *A*_mass_ and *R*_mass_, and low LMA. At the slow-return end of the spectrum, species had high LMA, low nutrient contents, and low *A*_mass_ and *R*_mass_ (Figure 2). Many other studies have reported that trends in the LES apply equally well to species growing in resource-poor environments. For example, Liu et al. (2010) found that correlated pairs of leaf and root traits held equally well for species adapted to semi-arid to arid environments. Correlations among leaves traits in forest understory ferns have also been shown to hold true for patterns reported for seed plants (Karst and Lechowicz, 2006). Therefore, resource availability appears to have only modest effects on LES trends (Wright et al., 2004).

It is noteworthy that sun and shade leaves do not depart from each other on the PC1 axis (Figure 2), indicating that trait plasticity does not change LES relationships between the two leaf-types, thereby supporting the expectations of the leaf-level carbon economics theory (Chabot and Hicks, 1982; Blonder et al., 2011; Michaletz et al., 2015, 2016). In contrast, Zhao et al. (2016) found that sun-acclimated species had a greater capacity to transport nutrients and higher rates of resource acquisition, whereas the reverse was true for shade- acclimated species manifesting slow-growth strategies. It is understandable that the economics strategies of sun and shade leaves will differ among different plant functional groups, such as tree and shrub species. Ordoñez et al. (2010) have demonstrated that qualitative attributes, such as growth form, woodiness, and leaf habit, can differ in accord with different environmental factors. In this context, our data show that deciduous and evergreen species cluster on the opposing ends of the LES likely because of their different acquisition strategies (Supplementary Table S4).

The LES does not consider the effects of within-canopy light availability on whole-plant performance. It implicitly assumes that within-canopy light gradients do not alter general scaling relationships. Our analyses support this supposition. The data show that the numerical values of the scaling exponents of key LES relationships are not statistically different between sun and shade leaves (Table 4 and Figure 3). For example, the scaling exponents for the *A*_mass_ vs. *N*_mass_ are numerically indistinguishable in sun and shade leaves (with a common slope of 1.69, 95% CIs = 1.48 and 1.93, *P* > 0.05). These similarities indicate that the LES predicts coordinated shifts among traits due to the fundamental biological trade-offs that underpin the LES and constrains leaf phenotypic plasticity across the subtropical forest canopy light gradients examined in this study (Reich et al., 2003; Shipley et al., 2006). Specifically, differences in species-level responses to changes in light conditions may not be sufficiently different to alter the relative positions of species along trait axes. This finding is consistent with the study of Keenan and Niinemets (2016) who report that the scaling relationships between LMA and both *A*_mass_ and *N*mass are similar in sun and shade leaves (see Figure 3 in Keenan and Niinemets, 2016). Furthermore, the scaling exponents between key leaf functional traits of shade and sun leaves do not differ numerically, even when evergreen and deciduous woody species are examined separately (Supplementary Table S4).

The numerical values of the scaling exponents for each of the leaf morphology vs. physiological scaling relationship in our study are all numerically larger than those in the world-wide data set, and fall within the lower region of the world-wide pattern of LMA (Table 4 and Figure 3), which consists of data drawn from plants growing at comparatively high temperatures in lower latitudes (Poorter et al., 2010). For the world-wide sun leaf dataset, the scaling relationship for LMA vs. *N*_mass_ (Table 4) is negatively allometric, i.e., a = -1.28 (95% CIs = -1.32 and 1.24 (Wright et al., 2004). Extensive N deposition tends to increase leaf N content (Peñuelas et al., 2015), which could explain the numerical difference observed between our data and those using the world-wide data set. The scaling exponents of the *N*_mass_ vs. *P*_mass_ scaling relationship for sun and shade leaves (i.e., 0.77 and 0.78, respectively) are similar to those reported by Niklas and Cobb (2005) who report a leaf *N*_mass_ vs. *P*_mass_ scaling exponent approximately equal to 0.75.

However, the scaling exponent of *N*_mass_ vs. *P*_mass_ in this study is numerically greater than that in the world-wide data set (with a slope 0.66) (Table 4 and Figure 3). A number of factors may help to explain this difference, particularly since our data are drawn from species living in a subtropical community. For example, Tian et al. (2017) report large variations in leaf *N*_mass_ vs. *P*_mass_ scaling relationships across latitudinal zones, wherein scaling exponents tend to numerically increase from boreal to tropical regions. In addition, the growing season and leaf longevity are longer in subtropical than in boreal regions, and leaf growth rates and P-demands are greater during the growing season with increasing latitude (Yan et al., 2017). Furthermore, soil P availability relative to N availability tends to increase with increasing latitude or from humid to arid regions, resulting in decreasing leaf N:P ratios (Han et al., 2005). These relationships help to explain at least in part why the leaf *N*_mass_ vs. *P*_mass_ scaling exponent reported here is numerically greater than that observed using a world-wide spectrum of data.

The N:P ratio in our study was also higher (18.37 ± 0.36) than that reported by Han et al. (2005) (i.e., N:P = 14.4) for plants across China, or He et al. (2008) for temperate and cold grasses in North China (i.e., N:P = 15.3). Most studies have concluded that P rather than N is the main factor limiting plant growth in the subtropical region (e.g., Reich et al., 1997; Han et al., 2005), which is consistent with the higher N:P ratio observed for the leaves examined in our study. This is also consistent with the larger scaling exponents observed for the LMA and *A*_mass_ vs. *P*_mass_ relationships (Table 4 and Figure 3), and the lower *R*_mass_ reported here compared to those for the world-wide data set (Table 4 and Figure 3).

Of particular interest in this regard is the observation that, although vastly different canopy irradiance gradient exhibit similar scaling exponents for any given trait relationship, the numerical values of the normalization constants of LES relationships differ significantly (Figure 3). These results may reflect quantifiable differences in species evolutionary and individual life histories (e.g., light) (Niklas and Hammond, 2019). Although the normalization constant is as biologically meaningful as the scaling exponent, little attention has been paid to how or why the normalization constant differs across data sets, or how it changes during the course of evolution by natural selection (Niklas and Hammond, 2019). In our study, the differences in the normalization constants of traits between sun and shade leaves were positively correlated with the differences in their related plastic index (Figure 4). This suggests that the variations in normalization constants might be attributable to differences in trait plasticity. N was allocated to metabolic functions in sun leaves and then a leaf with very high net gas exchange characteristics was constructed. Whereas shade leaves appeared to show excess uptake of N, which could be used for future use or allocated for non-photosynthesis (Chapin, 1980; Ågren, 2008). Therefore, at any given *N*_mass_, *A*_mass_, and *R*_mass_ were higher in sun leaves than in shade leaves. Furthermore, the constants for *A*_mass_ vs. *R*_mass_ and leaf morphology vs. *N*_mass_ are not significantly different for sun and shade leaves for either evergreen or deciduous woody species (Supplementary Table S5), indicating that differences in normalization constants for *A*_mass_ vs. *R*_mass_ and leaf morphology vs. *N*_mass_ across species are likely caused by differences of leaf habits among species in our data set. It is important to note that this inferences is drawn based on data drawn from subtropical communities. Because our study deals with only a relatively small community, more studies in different biomes are needed to confirm the generality of our findings.

The effects of within-canopy light gradients on leaf traits have been incorporated into canopy process (and land surface) models to predict canopy carbon flux and forest production (e.g., Ryu et al., 2011; Bonan et al., 2012). However, only some dynamic global vegetation models concerned with photosynthesis at the leaf level explicitly or implicitly consider the effects of shading within-canopy (Prentice et al., 2007). Our data contribute to a general LES for both sun and shade leaves and can inform future attempts at modeling whole canopy processes, particularly attempts to couple leaf photosynthesis and position within-canopy.

## Conclusion

Although the light availability gradient throughout canopies significantly affects physiological and morphological leaf traits, the scaling exponents of pairwise functional trait relationships for five mass-based leaf traits in sun and shade leaves do not numerically differ. These results support the proposition that a “canonical” LES exists and can be applied to shade leaves. Future progress toward understanding the scaling relationships elucidated by the LES nevertheless requires additional data with which to assess the effects of environmental factors on leaf traits, because it is becoming increasingly clear that the LES relationships are dependent on the species composition of communities.

## Data Availability Statement

The datasets generated for this study are available on request to the corresponding author.

## Author Contributions

XC, MW, QZ, and DC designed the research. XC, JS, and ML performed the research and analyzed the data. XC, MW, KN, SM, and DC wrote the manuscript. All authors read and approved the final manuscript.

## Conflict of Interest

The authors declare that the research was conducted in the absence of any commercial or financial relationships that could be construed as a potential conflict of interest.
