# Supplementary material for: The Leaf Economics Spectrum Constrains Phenotypic Plasticity Across a Light Gradient
Source: Front Plant Sci. 2020 Jun 11;11:735. doi: 10.3389/fpls.2020.00735 (PMC7300261; doi:10.3389/fpls.2020.00735)
Supplement: Supplementary file 1 [file Data_Sheet_1.docx]

**Supporting Information**

**

**

**Figure S1.** Histogram with normal distribution of photosynthetic photon flux densities (PPFDs) and light saturation point (LSP) of sun and shade leaves from the same canopy. (A): shade PPFDs as a percentage of sun PPFDs; (B): The photosynthesis (*A*_area_: area based photosynthesis)-light response curve; (C): Light saturation point of sun leaves; (D): Light saturation point of shade leaves. Different letters indicate significant differences in mean plasticity among traits (*t*-test, *P* < 0.05)





**Figure S2** Variance decomposition of five leaf economics spectrum traits including leaf mass per area (LMA), assimilation and respiration rates per unit mass (*A*_mass_ and *R*_mass_), mass-based nitrogen (N) and mass-based phosphorus (P) across different taxonomic scales.

**Table S1.** Classification of the 75 sampled tree species into family.

| Family | Genus | Species |
| --- | --- | --- |
| Anacardiaceae | Choerospondias | *Choerospondias axillaris* |
|  | Rhus | *Rhus chinensis* |
|  | Toxicodendron | *Toxicodendron succedaneum* |
| Aquifoliaceae | Ilex | *Ilex purpurea* |
|  |  | *Ilex elmerrilliana* |
| Araliaceae | Aralia | *Aralia chinensis* |
|  | Dendropanax | *Dendropanax dentiger* |
| Betulaceae | Betula | *Betula luminifera* |
| Calycanthaceae | Chimonanthus | *Chimonanthus nitens* |
|  |  | *Chimonanthus praecox* |
| Cornaceae | Dendrobenthamia | *Dendrobenthamia angustata* |
| Daphniphyllaceae | Daphniphyllum | *Daphniphyllum macropodrum* |
| Ebenaceae | Diospyros | *Diospyros morrisiana* |
|  |  | *Diospyros kaki* |
| Elaeocarpaceae | Elaeocarpus | *Elaeocarpus japonicus* |
|  |  | *Elaeocarpus sylvestris* |
|  | Sloanea | *Sloanea sinensis* |
| Ericaceae | Rhododendron | *Rhododendron simsii* |
| Euphorbiaceae | Mallotus | *Mallotus japonicus* |
|  | Sapium | *Sapium discolor* |
| Fabaceae | Albizia | *Albizia macrophylla* |
|  | Dalbergia | *Dalbergia hupeana* |
| Fagaceae | Castanea | *Castanea mollissima* |
|  | Castanopsis | *Castanopsis tibetana* |
|  |  | *Castanopsis fargesii* |
|  |  | *Castanopsis sclerophylla* |
|  |  | *Castanopsis carlesii* |
|  |  | *Castanopsis eyrei* |
|  | Cyclobalanopsis | *Cyclobalanopsis glauca* |
|  | Fagus | *Fagus longipetiolata* |
|  | Lithocarpus | *Lithocarpus glaber* |
|  | Quercus | *Quercus serrata* |
| Hamamelidaceae | Altingia | *Altingia gracilipes* |
|  | Liquidambar | *Liquidambar formosana* |
|  | Loropetalum | *Loropetalum chinense* |
| Illiciaceae | Illicium | *Illicium henryi* |
| Juglandaceae | Engelhardia | *Engelhardia fenzelii* |
|  | Platycarya | *Platycarya strobilacea* |
| Lauraceae | Cinnamomum | *Cinnamomum jensenianum* |
|  |  | *Cinnamomum camphora* |
|  | Lindera | *Lindera chienii* |
|  | Litsea | *Litsea cubeba* |
|  | Machilus | *Machilus thunbergii* |
|  |  | *Machilus leptophylla* |
|  |  | *Machilus velutina* |
|  | Phoebe | *Phoebe bournei* |
|  |  | *Phoebe faberi* |
| Magnoliaceae | Magnolia | *Magnolia denudata* |
|  | Manglietia | *Manglietia yuyuanensis* |
|  | Michelia | *Michelia skinneriana* |
| Meliaceae | Melia | *Melia azedarach* |
| Myricaceae | Myrica | *Myrica rubra* |
| Myrtaceae | Syzygium | *Syzygium buxifolium* |
| Nyssaceae | Nyssa | *Nyssa sinensis* |
| Rosaceae | Amygdalus | *Amygdalus persica* |
|  | Armeniaca | *Armeniaca mume* |
|  | Cerasus | *Cerasus discoidea* |
| Rubiaceae | Diplospora | *Diplospora dubia* |
|  | Randia | *Randia cochinchinensis* |
| Salicaceae | Populus | *Populus canadensis* |
|  |  | *Populus euramevicana* |
| Saxifragaceae | Itea | *Itea chinensis* |
| Scrophulariaceae | Paulownia | *Paulownia kawakamii* |
| Simaroubaceae | Ailanthus | *Ailanthus altissima* |
| Staphyleaceae | Euscaphis | *Euscaphis japonica* |
| Styracaceae | Alniphyllum | *Alniphyllum fortunei* |
|  | Styrax | *Styrax grandiflorus* |
| Symplocaceae | Symplocos | *Symplocos stellaris* |
|  |  | *Symplocos sumuntia* |
| Theaceae | Adinandra | *Adinandra millettii* |
|  | Camellia | *Camellia oleifera* |
|  | Eurya | *Eurya muricata* |
|  | Schima | *Schima superba* |
|  | Ternstroemia | *Ternstroemia gymnanthera* |
| Ulmaceae | Celtis | *Celtis sinensis* |

**Table S2.** Characteristics of sun and shade branches from 75 tree species in a subtropical forest.

| Shoot traits | Sun branches | | Shade branches | |
| --- | --- | --- | --- | --- |
|  | Range | Mean ± se | Range | Mean ± se |
| Diameter (mm) | 0.60-7.12 | 1.91 ± 0.04 | 0.59-4.79 | 1.80 ± 0.03 |
| Length (mm) | 24.55-212.40 | 81.49 ± 2.11 | 21.68-181.44 | 73.04 ± 1.95 |
| Cross-sectional area (mm^2^) | 0.30-40.06 | 3.54 ± 0.19 | 0.28-18.91 | 3.07 ± 0.12 |
| Volume (mm^3^) | 26.12-1267.75 | 272.23 ± 14.1 | 25.34-1577.71 | 219.88 ± 12.5 |
| Density (mg mm^-3^) | 0.29-2.97 | 0.98 ± 0.06 | 0.36-3.80 | 1.21 ± 0.07 |
| Mass (mg) | 18.08-639.98 | 134.05 ± 6.71 | 16.28-978.85 | 148.62 ± 7.00 |

There were no significant differences between sun- and shade branches for any of the measured traits (all *P* >0.05).

**Table S3.** Summary of traits for sun and shade leaves across 61 genera and 33 families in a subtropical forest.

| Taxonomic scales | Traits | Sun leaves | Shade leaves | Plasticity |
| --- | --- | --- | --- | --- |
| Genus | LMA (g m^-2^) | **81.03 ± 3.40*** | **71.65 ± 2.64*** | **1.15 ±0.04^c^** |
|  | *A*_mass_ (nmol g^-1^ s^-1^) | **119.51 ± 7.35*** | **69.52 ± 4.26*** | **1.82 ± 0.08^a^** |
|  | *R*_mass_ (nmol g^-1^ s^-1^) | **10.00 ± 0.84*** | **6.56 ± 0.51*** | **1.59±0.09^b^** |
|  | *N*_mass_ (%) | 2.06 ± 0.08 | 2.02 ± 0.07 | **1.02 ± 0.01^d^** |
|  | *P*_mass_ (%) | 0.11 ± 0.004 | 0.12 ± 0.005 | **0.98 ± 0.02^d^** |
| Family | LMA (g m^-2^) | **78.43 ± 3.48** | **70.34 ± 2.93** | **1.14 ±0.05^c^** |
|  | *A*_mass_ (nmol g^-1^ s^-1^) | **124.13 ± 9.83*** | **73.18 ± 5.49*** | **1.75 ± 0.09^a^** |
|  | *R*_mass_ (nmol g^-1^ s^-1^) | **10.66 ± 1.11*** | **7.00 ± 0.70*** | **1.63 ± 0.12^b^** |
|  | *N*_mass_ (%) | 2.07 ± 0.10 | 2.03 ± 0.09 | **1.02 ± 0.01^d^** |
|  | *P*_mass_ (%) | 0.11 ± 0.01 | 0.12 ± 0.01 | **0.99 ± 0.02^d^** |

Significant differences are denoted in bold; asterisks indicate significant differences in trait means between sun and shade leaves, while superscripted letters indicate significant differences in mean plasticity among traits (*t*-test, *P* < 0.05).

**Table S4.** Summary of traits for different growth forms and leaf habits in a subtropical forest.

| Traits | Growth forms | | Leaf habits | |
| --- | --- | --- | --- | --- |
|  | Shrub | Tree | Deciduous | Evergreen |
| LMA (g m^-2^) | 81.11 ± 3.71 | 78.42 ± 2.48 | **63.14 ± 2.10*** | **90.96 ± 2.64*** |
| *A*_mass_ (nmol g^-1^ s^-1^) | 89.65 ± 6.89 | 90.46 ± 4.21 | **121.23 ± 7.02*** | **68.25 ± 3.74*** |
| *R*_mass_ (nmol g^-1^ s^-1^) | 6.72 ± 0.56 | 8.60 ± 7.88 | **12.32 ± 0.83*** | **4.74 ± 0.24*** |
| *N*_mass_ (%) | 1.90 ± 0.09 | 2.09 ± 0.05 | **2.42 ± 0.06*** | **1.73 ± 0.05*** |
| *P*_mass_ (%) | 0.11 ± 0.01 | 0.12 ± 0.004 | **0.14 ± 0.004*** | **0.09 ± 0.003*** |
| PC1 score | -0.37 ± 0.03 | 0.26 ± 0.03 | **0.07 ± 0.06*** | **-0.04 ± 0.04*** |

Significant differences are denoted in bold; asterisks indicate significant differences in trait means between sun and shade leaves, while superscripted letters indicate significant differences in mean plasticity among traits (*t*-test, *P* < 0.05).

**Table S5.** Bivariate relationships among mass-based leaf traits of evergreen and deciduous species.

| Habits | Leaf traits | log LMA | log *A*_mass_ | log *N*_mass_ | log *P*_mass_ | log *R*_mass_ |
| --- | --- | --- | --- | --- | --- | --- |
| Evergreen species | log LMA |  | -0.66 (-0.79,-0.55) | -1.02 (-1.21, -0.86) | -0.93 (-1.12, -0.77) | -0.60 (-0.70, -0.51) |
|  | log *A*_mass_ | *** |  | 1.54 (1.28, 1.86) | 1.40 (1.15, 1.70) | 0.90 (0.76, 1.08) |
|  | log *N*_mass_ | ns | *** |  | 0.91 (0.78, 1.05) | 0.58 (0.49, 0.70) |
|  | log *P*_mass_ | ns | *** | ns |  | 0.64 (0.53, 0.78) |
|  | log *R*_mass_ | *** | ns | *** | *** |  |
| Deciduous species | log LMA |  | -0.68(-0.87,-0.54) | -1.37 (-1.78, -1.06) | -0.95 (-1.24, -0.73) | -0.57 (-0.72, -0.45) |
|  | log *A*_mass_ | *** |  | 2.00 (1.52, 2.62) | 1.39 (1.07, 1.80) | 0.84 (0.65, 1.08) |
|  | log *N*_mass_ | ns | *** |  | 0.69 (0.55, 0.87) | 0.42 (0.32, 0.55) |
|  | log *P*_mass_ | ns | *** | ns |  | 0.61 (0.47, 0.79) |
|  | log *R*_mass_ | *** | ns | * | *** |  |

The common standardized major axis exponents with 95% confidence intervals for the sun and shade leaves are given in the lower left section of the matrix (*y* variable is column 1, *x* variable in row 1). * indicates normalization constants were differed significantly (*P* <0.05); ** indicates normalization constants were differed significantly (*P* <0.01); *** indicates normalization constants were differed significantly (*P* <0.001).
